# Fabrication and Performance of Continuous 316 Stainless Steel Fibre-Reinforced 3D-Printed PLA Composites

**DOI:** 10.3390/polym16010063

**Published:** 2023-12-24

**Authors:** Alison J. Clarke, Andrew Dickson, Denis P. Dowling

**Affiliations:** 1 I-Form Centre, School of Mechanical & Materials Engineering, University College Dublin, Belfield, D04 C1P1 Dublin, Ireland; denis.dowling@ucd.ie; 2 Infraprint, Nova UCD, Belfield, D04 C1P1 Dublin, Ireland; andrew.dickson@infraprint.com

**Keywords:** 3D printing, thermoplastic polymers, mechanical properties

## Abstract

This study investigates the feasibility of 3D printing continuous stainless steel fibre-reinforced polymer composites. The printing study was carried out using 316L stainless steel fibre (SSF) bundles with an approximate diameter of 0.15 mm. This bundle was composed of 90 fibres with a 14 μm diameter. This fibre bundle was first coated with polylactic acid (PLA) in order to produce a polymer-coated continuous stainless steel filament, with diameters tailored in the range from 0.5 to 0.9 mm. These filaments were then used to print composite parts using the material extrusion (MEX) technique. The SSF’s volume fraction ($V_{f}$) was controlled in the printed composite structures in the range from 4 to 30 $V_{f}$%. This was facilitated by incorporating a novel polymer pressure vent into the printer nozzle, which allowed the removal of excess polymer. This thus enabled the control of the metal fibre content within the printed composites as the print layer height was varied in the range from 0.22 to 0.48 mm. It was demonstrated that a lower layer height yielded a more homogeneous distribution of steel fibres within the PLA polymer matrix. The PLA-SSF composites were assessed to evaluate their mechanical performance, volume fraction, morphology and porosity. Composite porosities in the range of 2–21% were obtained. Mechanical testing demonstrated that the stainless steel composites exhibited a twofold increase in interlaminar shear strength (ILSS) and a fourfold increase in its tensile strength compared with the PLA-only polymer prints. When comparing the 4 and 30 $V_{f}$% composites, the latter exhibited a significant increase in both the tensile strength and modulus. The ILSS values obtained for the steel composites were up to 28.5 MPa, which is significantly higher than the approximately 13.8 MPa reported for glass fibre-reinforced PLA composites.

## 1. Introduction

Additive manufacturing (i.e., 3D printing) enables components to be fabricated by adding material layer by layer. Several 3D printing materials are available to fabricate components including polymers, metals, ceramics and composites [1,2,3,4,5,6,7,8,9,10,11,12]. Polymer components which are 3D printed exhibit relatively poor mechanical performance compared with those obtained by injection molding, and hence the interest in adding reinforcing materials [13]. This study investigates the use of a continuous steel fibre bundle as reinforcement for 3D-printed polymer composites.

One of the most widely used 3D printing techniques is material extrusion (MEX) [14,15,16]. Polymer composites are fabricated through the addition of fibres (short or continuous) or alternatively powder particles, beads and pellets [13,17,18,19,20]. The reinforcing fibres available for MEX-printed composite reinforcing fibres are vast and include glass, metal, carbon and basalt [21,22,23]. The most commonly used thermoplastic feedstocks include polylactic acid (PLA), polycarbonate (PC), polyamide (PA or nylon) and acrylonitrile butadiene styrene (ABS) [13,16,17,18,19,20,24].

The two approaches which are routinely used to fabricate composites through MEX are called ex situ prepreg or in situ fusion [25,26,27]. The latter method is the one most widely used for the fabrication of continuous fibres and involves direct fibre integration into the print nozzle during 3D printing [12,13,28,29]. Ex situ prepreg production is a two-part process involving fabricating the composite filament and then using the composite filament in the 3D printing process [13]. In situ fusion provides a more rapid route to fabricating composite parts compared with ex situ prepreg [30]. However, due to the short dwell time, along with insufficient pressure from the nozzle and large temperature gradients, there can be issues with poor infusion and adhesion of the polymer matrix around the fibres [27,31]. An example of the ex situ prepreg process was investigated by Hu et al. [27] for the printing of PLA and continuous carbon fibre filaments. Chen et al. [32] also used the ex situ prepreg method to 3D print PLA containing continuous glass fibre (PLA-cGF). It was reported that there was a high level of impregnation of the PLA between and into the fibres.

Heidari-Rarani et al. [33] reported on the development of an in situ fusion 3D printer extruder head designed to reduce nozzle blockage during the fabrication of filaments of PLA reinforced with continuous carbon fibre (PLA-cCF). To improve PLA and cCF bonding, the fibres were pretreated or sized with a PVA solution, resulting in composite parts with a volume fraction of 28.2% and void content of 9.1%. Li et al. [34] also investigated the 3D printing of PLA-cCF, including the use of PLA sizing agents on the fibres. The continuous carbon fibre bundle contained up to a maximum of 1000 individual fibres. The resulting composite exhibited a volume fraction ($V_{f}$) of 34% in a unidirectional printing pattern, along with a tensile strength of up to 91 MPa. Rimašauskas et al. [35] investigated using polymer solutions of PLA, PC and ABS for pre-preg sizing to dry cCF at different concentrations by using in situ fusion 3D printing with respective 1.75 mm filaments. A 10% pre-preg concentration resulted in a 25.2 $V_{f}$% and tensile strength of 165 MPa. Maqsood et al. [36] used the in situ fusion method along with the PLA pre-impregnated cCF tow to reinforce the PLA (PLA-cCF) and a cCF tow to reinforce the PLA containing short carbon fibres (PLA-SCF-cCF). The resultant composites exhibited 18.5 $V_{f}$% and 21.8 $V_{f}$% respectively. The composite with the PLA-continuous carbon fibre exhibited a tensile strength of up to 245.4 MPa, but the PLA-SCF-cCF composite was lower at 227.6 MPa.

Caminero et al. [37] evaluated the effects on the interlaminar bonding due to layer thickness and various fibre volume fractions of 3D-printed nylon reinforced with continuous fibres of Kevlar, glass and carbon supplied by Markforged. The interlaminar shear strength was evaluated for nylon-Kevlar, nylon-glass and nylon-CF, with resultant strengths of 14.3, 21.0 and 31.9 MPa, respectively. The fibre content plays an important role in determining the properties of MEX composite filaments, such as with the tensile strength generally increasing with increasing fibre content [13]. One difficulty, however, is that composite filaments, with a high fibre content, can be quite difficult to print, arising from issues with nozzle clogging, in addition to the excessive viscosity of the melted composite filament [24,33,38,39,40,41,42].

There have been very few reports on the incorporation of continuous steel fibres into 3D-printed composites. Quan et al. [43] evaluated the use of a laminating approach, in which an epoxy sandwich containing SSF with carbon fibre was fabricated. The addition of the steel fibre was reported to yield a significant increase in interlaminar and fracture toughness compared with carbon fibre-reinforced plastics. Ibrahim et al. [18,30] and Saleh et al. [19] successfully 3D printed continuous wire polymer composites for sensor applications. These authors used in situ fusion to combine 75 μm nickel-chromium wires and 75 μm copper wire with PLA filament. Only a small increase in the mechanical properties of the PLA was reported through incorporation of the metal wires.

Hamidi et al. [44] investigated 3D printing PLA and bright finish conductive copper wire, as well as the polymer combined with spring-back 302/304 stainless steel wire with diameters of 0.130 mm and 0.178 mm, respectively, as reinforcement for bioinspired joint fabrication. Both samples were printed using in situ fusion with filament 1.75 mm in diameter. It was reported that the copper wire broke when printed in a concentric pattern due to the traction force being too large. Increasing the copper wire’s $V_{f}$ above 0.4% resulted in little improvement in the tensile modulus, and the steel wire showed poor adhesion and bonding with the PLA during tensile testing. Gunes et al. [45] evaluated the tensile strength of nylon reinforced with continuous stainless steel wire (50 μm in diameter) and demonstrated an increase in nylon tensile strength of 7.6 times when 3D printed in a concentric pattern.

The focus of the current study is to investigate the feasibility of printing continuous SSF-reinforced polymer composites. A particular focus of the investigation is to achieve a high fibre volume fraction along with low porosity within the printed composite parts in order to enhance mechanical performance.

## 2. Materials and Methods

### 2.1. Materials

**Figure 1:**
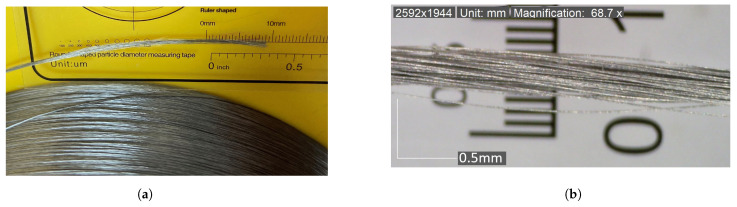
Stainless steel fibre bundle: (**a**) spool and (**b**) 90 fibres per bundle.

The starting points for this study involved the fabrication of stainless steel-reinforced polymer filaments. A continuous 316 L stainless steel fibre (SSF) bundle (Figure 1a) was obtained from NV Bekaert SA (Brussels, Belgium) [31,46]. The SSF was fabricated with a `bundle wire drawing’ manufacturing process, which resulted in a hexagonal fibre cross-section [47,48]. The fibre bundle had a diameter of approximately 0.15 mm and consisted of 90 fibres per bundle, each with a diameter of 14 μm. The bundle had a linear density of 110 decitex (TEX), torsion per cm of 1 and Young’s modulus of 200 GPa [46]. The elemental composition of the steel fibre was determined using energy-dispersive X-ray spectroscopy (EDAX), and the results are detailed in Table 1.

The PLA polymer used to coat the steel to form the filament was obtained as pellets from Nature Works, with a product reference of Ingeo${}^{\mathrm{TM}}$ Biopolymer D4043D [1]. The PLA pellets were dried at 55 °C for a minimum of 24 h prior to extrusion.

#### 2.1.1. PLA-Reinforced SSF Filament

**Figure 2:**
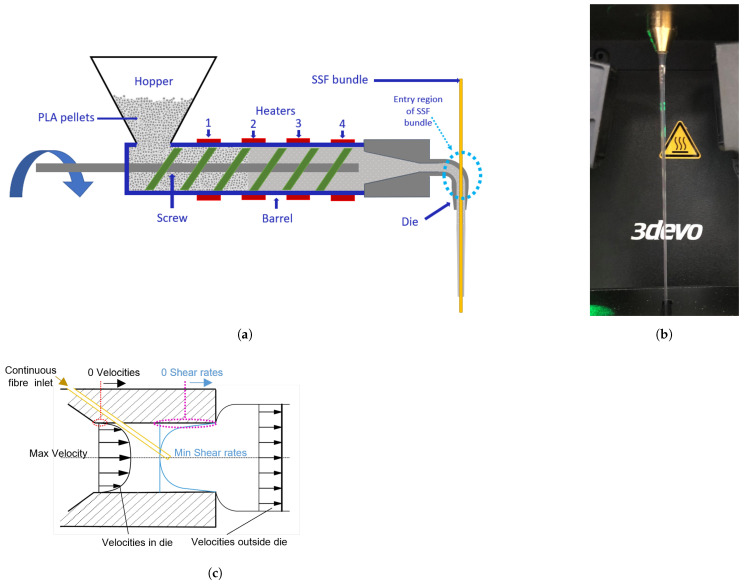
Schematic of 3devo filament making. (**a**) Schematic with the fibre introduction region indicated by the blue dashed ovals. (**b**) Photograph of the PLA-SSF filament co-extrusion. (**c**) Velocity and shearing profile of a non-Newtonian polymer-fibre co-extrusion die.

The fabrication of a PLA-SSF filament was carried out using a 3devo laboratory-scale filament maker (Utrecht, Netherlands) [49]. This was modified to facilitate the introduction of the fibre into the molten polymer during filament extrusion, as shown in Figure 2. The SSF was introduced into the molten polymer using a custom-designed extrusion die similar to those described in the literature [50,51,52]. The die design minimised the forces on the fibre bundle during co-extrusion by helping to entrain the fibre bundle in the polymer flow. As highlighted by previous authors, in this die design, where the fibre bundle enters the polymer flow, there is a region of maximum velocity, with the lowest inter-material shearing and pressure as shown in Figure 2c [53,54,55,56,57]. As the SSF bundle travels through the die, it undergoes preheating before entering the molten polymer, decreasing the temperature gradient between the materials [58].

Fans cool the polymer-coated SSF bundle after extrusion. We note that rapid cooling can cause polymer melt fracturing or partially solidify the polymer in the extrusion die, resulting in teardrop shapes forming along the SSF [51]. DevoVision-win32-v0.2.0, used in conjunction with the 3devo filament maker, recorded the filament diameter after the cooling zone and before spooling. It was observed that a reduction in the filament diameter below 0.4 mm was indicative of issues, such as poor polymer adherence to the SSF.

The 3devo filament maker processing parameters used for fabrication of the PLA-SSF filaments are given in Table 2. In order to achieve lower layer heights, it was found to be necessary to decrease the filament diameters from 0.9 to 0.5 mm, as demonstrated in Table 2. Reducing the extrusion speed slowed the extrusion barrel revolution and velocity of the polymer entering and exiting the die.

#### 2.1.2. Three-Dimensional Printing Continuous PLA-SSF Composite Components

**Figure 3:**
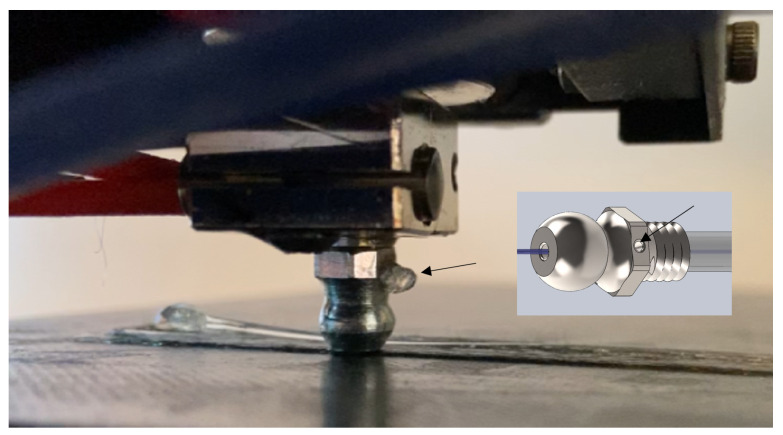
Photograph of the printing head with an insert showing a schematic of the 1 mm in diameter polymer pressure vent (arrow), which facilitated the removal of excess polymer during printing. Note also the 4.5 mm flat area on the print head, which was found to facilitate ‘ironing’ of the part during printing.

The 3D printing using the PLA-SSF filaments was carried out using a modified Anycubic i3 Mega polymer extrusion printer (Shenzhen, China). The modifications included design change innovations to the polymer-continuous fibre printing head nozzle in order to facilitate the removal of *‘excess’* polymer during printing. This was achieved by incorporating a *‘polymer pressure vent’* into the print head nozzle, as shown in Figure 3. Excess polymer around the continuous stainless steel wire was allowed to exit through this nozzle 1 mm in diameter during printing. This excess polymer release vent was positioned at the end of the hot zone in the print head as at this location, the polymer was likely to exhibit a lower viscosity. The polymer material was routinely removed during printing to prevent it from falling onto the print surface. As the distance from the print head to the substrate was reduced, there was a change in the level of pressure in the print head, which was relieved by the removal of excess polymer through the polymer pressure vent. Thus, the rate of removal of excess polymer was directly related to the print layer height, with higher levels of removal occurring at lower heights. This thus facilitated control of the metal fibre content within the printed composites. One of the advantages of the pressure release outlet was that it helped to reduce filament failure during printing. By overcoming the backpressure, it was also found to improve the composite surface finish.

An additional printer design innovation is the flattened print head illustrated in Figure 3. This 4.5 mm in diameter ‘flat’ nozzle head appeared to have an ‘ironing’ effect on the print, resulting in an increase in the head dwell time during printing, which should facilitate greater polymer diffusion between the fibres in the steel bundle. A further modification was the addition of a soft-wheeled filament feeder, and this was found to help reduce the level of continuous filament breakage during printing.

The print geometries were created using a computer-aided design (CAD) file (Solidworks 2021 software, version 23.3.0.0059,) and then exported as a stereolithography (STL) file. The open-source slicing software PrusaSlicer v.2.6.1 was used to slice the STL file generating the G-code for 3D printing. All test samples were 3D printed in a unidirectional continuous pattern (0°). To achieve a continuous printing path with no cutting of the fibres between layers or at the end of each path, only perimeters were selected in the slicing software v.2.6.1.

Printing at layer heights below the PLA-SSF filament diameter (minimum of 0.5 mm) meant there was an excess of polymer in the system, as the maximum quantity of polymer on the print bed was deposited. The excess polymer was either deposited on the surface, resulting in a poor surface finish, or returned up the printing nozzle, generating backpressure and clogging.

**Figure 4:**
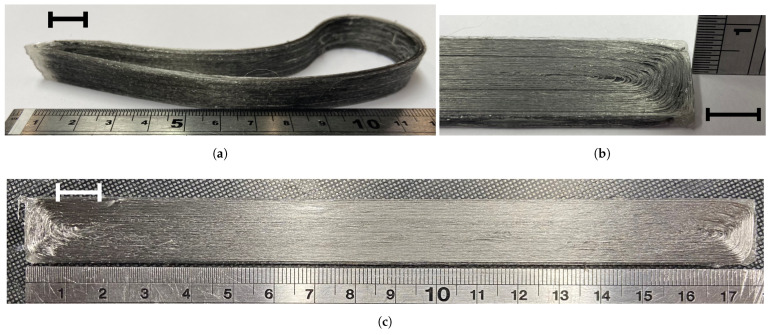
Three-dimensional printing continuous PLA-SSF composite. (**a**) Geometry shape trial prints. (**b**) Close-up of PLA-SSF continuous 3D printing path. (**c**) Tensile sample printed at a layer height of 0.22 mm, (10 mm scale bar).

A variety of processing parameters and geometry modifications were optimised. This included adjusting the temperatures of the print head heating, print bed and cooling to manage temperature gradients. Additionally, the printing path height and hatch spacing (Figure 4a) were altered, and the deposition speeds of the perimeters and between layers were varied. The print heights were varied between 0.48 and 0.22 mm. In the case of the lowest layer height of 0.22 mm, the print head temperature was increased, as demonstrated in Table 3. The elevated temperature was required to reduce the viscosity of the polymer and to facilitate better flow of the molten polymer as the PLA-SSF exited the printing head. The higher temperature was also found to prevent the polymer from cooling prematurely and solidified within the printing head.

The relatively high thermal conductivity of the stainless steel was likely to assist the composite in retaining a temperature close to the bed temperature during printing, giving the SSF additional dwell time. Thus, the coupling of the composite filament feed rate with the deposition rate and the deposited material’s elevated temperature were likely to reduce internal stresses as well as warping.

Experimental 3D prints carried out with various print radii and angles demonstrated that the PLA-SSF filament could achieve filament turns of up to 180° without breakage or degradation of the fibres. An example is shown in Figure 4a. This is in contrast with the behaviour of continuous carbon fibre filaments, which were reported to fail when high filament turn angles are used [37].

Figure 4b shows a close-up at the end of a single tensile sample. Where each line of a continuous SSF bundle’s continuous turning path is displayed, a 180° turn is evident along the centre of the print due to the flexibility of the fibres in the bundles. In the case of the 90° corners, however, the `clear’ polymer evident at the right edge of the print indicates that it was not reinforced. The printing path executed the G-code to the corner, depositing the continuous PLA-SSF filament to the 90° corner. However, the polymer solidified at a slower rate than the print heads travelled, which resulted in the SSF drawing through the polymer curving around the corners. Additional cooling and reducing the print spread during cornering helped to minimise this effect in the non-reinforced region. This low turning angle ability of the stainless steel facilitated the direct printing of tensile samples (Figure 4c).

Based on varying a range of printing parameters, the optimised printing conditions were identified, and they are given in Table 3. It was observed that lower print layer heights generally yielded superior printed part performance, as observed previously by other authors [59]. The optimum obtained print layer height of 0.22 mm was similar to that recommended by manufacturers for commercial filaments of PLA reinforced with short carbon fibres [33]. An advantage of the ex situ prepreg approach is an increase in material cohesion.

### 2.2. Composite Characterisation

The print morphology was examined using an inverted metallurgical microscope (Olympus GX51, (Olympus Corporation, Tokyo, Japan) along with a Tabletop Hitachi tm1000 scanning electron microscope (SEM), (Hitachi High-Technologies Corporation, Tokyo, Japan, sourced from Hitachim UK). The specimen dimensions were obtained using Digi Plus Line digital vernier callipers.

#### 2.2.1. Polymer-SSF Filament

Optical and SEM microscopy were used to investigate the morphology, the encapsulation of the SSFs as well as the level of polymer impregnation between the SSFs. This was facilitated by mounting the composite filament in a two-part cold cure Polytek Easyflow resin. The cure temperature was below the glass transmission temperature of the polymer so as to not distort its structure. The samples were ground and polished with abrasive silica paper down to 2000 grit and then polished with a 3 μm diamond suspension.

#### 2.2.2. Three-Dimensional Printed Composites

The porosity and internal structure of the 3D-printed components was evaluated using a GE Nanotom X-ray micro computed tomography (μCT) scanner (GE Sensing & Inspection Technologies GmbH, Wunstorf, Germany), which has up to an 8 μm resolution. Analysis of the μCT scans was carried out using VG Studios software, version 3.5. The μCT scan results were cross-referenced with images obtained using microscopy. The latter images were imported for measurement using image processing software (ImageJ, National Institutes of Health, Bethesda, MD, USA, version Java 1.8.0_345). The volume fraction (V${}_{f}$) and the number of fibres in the PLA-SSF 3D-printed structure were determined from the μCT scans and ImageJ analysis. The volume fraction ($V_{f}$) was calculated using Equation (1) [60]:

(1) $$V_{f}\;\left(\%\right)=\frac{V_{SSF}}{V_{T}}$$

where: $V_{SSF}$ is the volume of the stainless steel fibres in the bundle and $V_{T}$ is the overall volume of the composite. The porosity and $V_{f}$ analysis used different threshold settings to identify the materials. The CT scan images included multiple cross-sections of the PLA-SSF filament obtained from at least three separate 3D-printed parts.

### 2.3. Mechanical Performance

The mechanical performance of the PLA-SSF composites was assessed based on both the interlaminar shear strength according to ASTM D2344 along with the tensile properties following ASTM D5082 [61,62]. Tests were conducted using Zwick Roell Z005 (The ZwickRoell Group, Ulm, Germany. Sourced from ZwickRoell Ltd., Worcester, UK) and Lloyd 6000S (AMETEK, Inc., Berwyn, PA, USA) mechanical testers with 10 and 30 kN load cells, respectively. The data analysis followed the guidelines outlined in ASTM D2344 and D5082 to determine the sample mean, standard deviation and coefficient of variation (expressed as a percentage).

#### 2.3.1. Interlaminar Shear Strength

The interlaminar shear strength (ILSS) was determined through short beam strength testing and was used to investigate the cohesion between different material combinations [61]. The short beam test was performed at a speed of 1 mm/min. The ILSS sample size was defined by the sample thickness (h), the width (b) was two times that thickness, and the length (l) was six times that thickness, with each measuring 3 × 6 × 18 mm. The span of the lower beams *S* was also defined by the thickness as 4 × (h), measuring 12 mm. The samples were cut to the length using a precision saw (Buehler Isomet${}^{\mathrm{TM}}$ High-Speed Pro (Bluff, IL, USA)) and shaped to the required dimensions using a water grinding wheel. The ILSS strength $\tau_{ILSS}$ (MPa) was calculated using Equation (2) [61]:

(2) $$\tau_{ILSS}=\frac{3}{4}\times \frac{P_{m}}{b\times h}$$

where $P_{m}$ is the maximum load observed or failure during the test (N), *b* is the specimen width (mm) and *h* is the specimen thickness (mm).

#### 2.3.2. Tensile Testing

The tensile test samples had dimensions of 3 × 15 × 175 mm. Sample preparation included grinding all sets to the correct dimensions and bonding steel tabs with dimensions of 1.5 × 15 × 56 mm with an angle of 30° to each end and side of the tensile samples using Loctite 480 Cyanoacrylate (Henkel Adhesive Technologies, Dusseldorf, Germany. Supplied by RS-Ireland. The tensile test was executed at a speed of 5 mm/min. The tensile strength and tensile modulus properties were determined. The PLA-SSF tensile strength ($F_{tu}$) was calculated using Equation (3) [62]:

(3) $$F_{tu}=\frac{P_{max}}{A}$$

where $P_{max}$ is the maximum force at failure and *A* is the cross-sectional area. A minimum of five test samples were evaluated under each test condition.

## 3. Results and Discussion

### 3.1. Filament Characterisation

**Figure 5:**
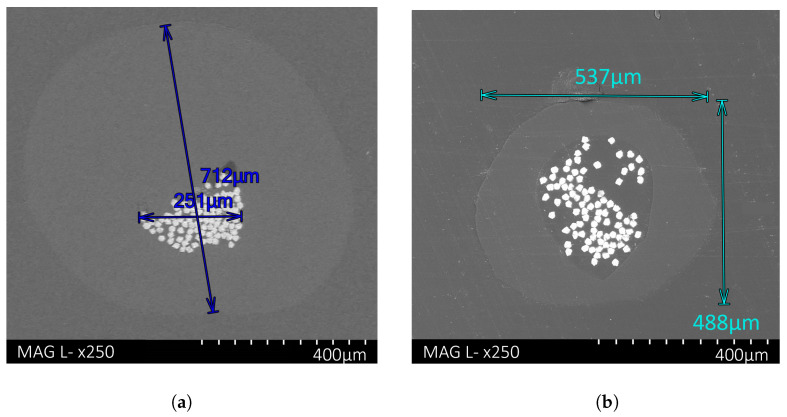
Cross-section SEM images of PLA-SSF filaments mounted in resin: (**a**) 0.7 mm PLA-SSF filament and (**b**) 0.5 mm PLA-SSF filament. Note the greater dispersion of fibres in the bundle for the filament with the smaller diameter.

The composite polymer-SSF filament morphology, cohesion and dimensions were captured using both optical and SEM microscopy. A cross-sectional SEM image of a PLA-SSF filament 0.7 mm in diameter is displayed in Figure 5a. The fibres were tightly grouped, and the bundle retained an ovular or circular cross-sectional shape, a similar shape to that before SSF’s addition to the polymer. The fibre’s hexagonal cross-section was a result of the bundle wire-drawing process, as each fibre’s flat side aligned with the next fibre, restricting the polymer diffusion to the inner fibres. The results indicated low levels of PLA impregnation within the bundle and porosity around the circumference of the bundle, indicating poor diffusion or adhesion between materials.

In contrast to the filament 0.7 mm in diameter, Figure 5b shows a 0.5 mm PLA-SSF filament cross-section. In this case, the fibres were dispersed across a wider area within the polymer matrix, with a high level of polymer diffusion through the fibres and a low level of porosity. In more commercial applications, the filament diameter can be reduced further.

### 3.2. Printed Composite Evaluation

The continuous steel fibre-reinforced composites’ morphology was first evaluated based on a combination of cross-sectional examination and μCT analysis. This was followed by an evaluation of the composite’s mechanical properties based on ILSS and tensile testing.

**Figure 6:**
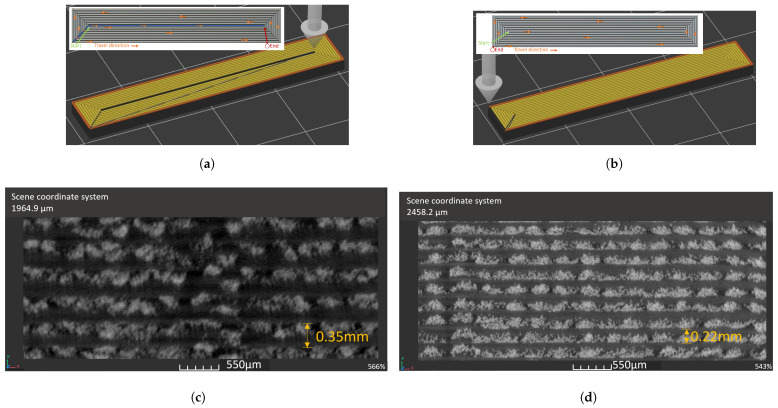
The μCT scans of PLA-SSF 3D-printed part cross-sections (scale bar = 550 μm). (**a**) Printing head travel path, with uneven perimeters of the 0.35 mm layer height samples. (**b**) Printing head travel path, with even perimeters of the 0.22 mm layer height samples. (**c**) Composite (12 $V_{f}$%) printed with layer height of 0.35 mm. (**d**) Composite (30 $V_{f}$%) printed with layer height of 0.22 mm.

Examples of μCT scan images of the 3D-printed composite sample cross-sections printed at layer heights of 0.35 and 0.22 mm are given in Figure 6c,d, respectively. The large number of fibres within each SSF bundle is clearly evident within the images, and their high surface areas led to enhanced contact between the fibres and the polymer. Note that the 0.22 mm print had a significantly enhanced stainless steel content along with a more homogeneous distribution of fibres. As detailed in Section 2.1.2, the higher steel fibre content for this composite was facilitated by the removal of the excess polymer through the ‘polymer pressure vent ’ nozzle on the print head.

As illustrated in Figure 6d, for the 0.22 mm layer height, the SSF in the composite structure indicates a well-ordered, relatively homogenous steel fibre arrangement achieved by increasing the geometrical dimensional widths by 0.4 mm. After structural analysis of the 0.35 mm sample set (Figure 6c), a distortion along the centre seam of the samples was observed. The distortion in the structure was due to an uneven number of printed concentric perimeters caused by the hatch spacing of 0.4 mm being an uneven multiple of the 0.35 mm sample’s designed width. The uneven printer head travel path is shown by the orange arrows in Figure 6a along with the blue line in the middle of the geometry. The G-code was compiled for uneven perimeters to print the start of each layer at 0.4 mm from the centre, generating a void (blue line in Figure 6a), and the last line of material deposited was in this space. It was assumed from the polymer 3D printing that there was no material in this region. However, the excess polymer in this polymer-SSF system flowed into the void from the first perimeter deposition and solidified. Obstructing the final line of material deposition caused a structural distortion. The adjustment to the CAD geometry altered the G-code to generate an even number of perimeters (Figure 6b), and this allowed the more homogeneous laying down of fibres in Figure 6d for the height of 0.2 mm. The samples were prepared for mechanical testing as outlined in Section 2.3.

**Figure 7:**
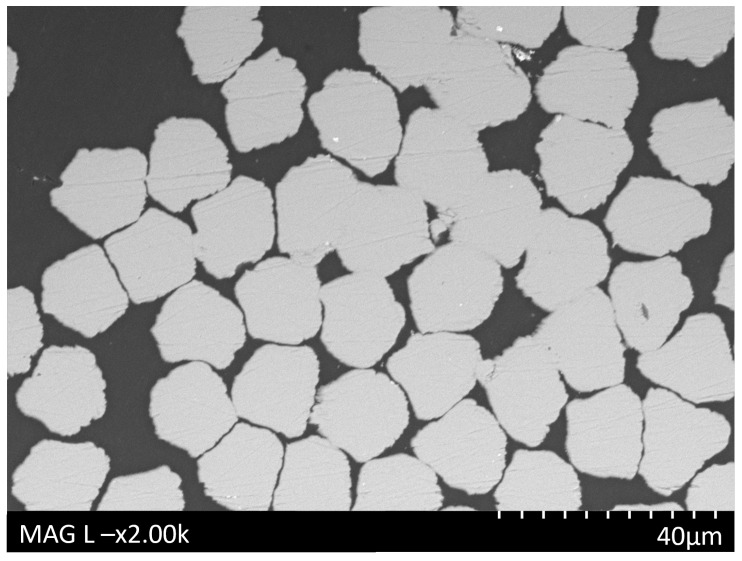
SEM cross-section image of the PLA-SSF composite matrix printed using a 0.22 mm layer height, demonstrating good diffusion of the PLA between the fibres in the SSF bundle.

SEM examination of the cross-section of the composite printed at a layer height of 0.22 mm demonstrated the high level of impregnation of the PLA into the steel fibre bundle (Figure 7).

The results for the μCT porosity are given in Table 4, which demonstrates that there was a significant reduction in porosity, as the print layer height was reduced between 0.45 and 0.22 mm. This reduction in porosity was similar to the results reported by other authors, being associated with layer height thickness reduction [20]. In addition to the layer height, other factors which contributed to the porosity reduction included improvements in the travel path, which assisted in creating a more symmetrical, ordered structure as outlined in Section 3.2. Associated with the print layer height reduction was an increase in the SSF content, with the $V_{f}$ increase being from 6 to 30%.

### 3.3. Mechanical Testing

#### 3.3.1. Interlaminar Shear Strength

**Figure 8:**
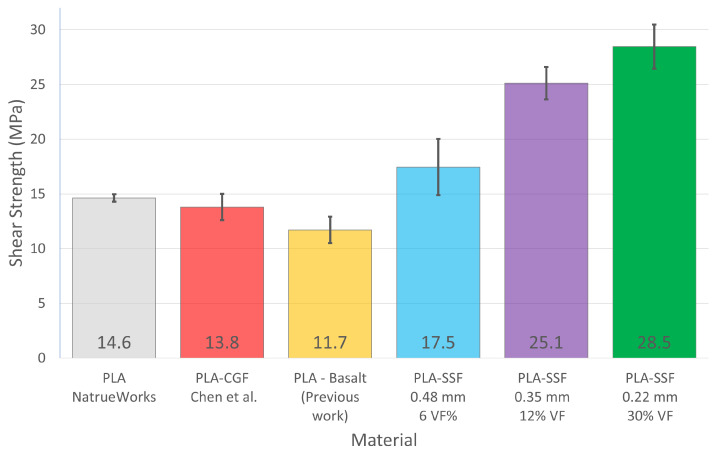
Interlaminar shear strength results for the PLA 0.22, 0.35 and 0.48 mm layer height prints, along with the results reported in the literature for PLA-glass fibres (cGF) and a PLA-Basalt composite investigated in a previous study at UCD (not reported) [32].

The results of the interlaminar shear strength ($\tau_{ILSS}$) tests for the four layer heights investigated are given in Figure 8. For comparison purposes, this graph includes reports from the literature for 3D-printed continuous glass fibre-reinforced PLA (PLS-cGF) composites reporting a $\tau_{ILSS}$ of 13.8 MPa (±1.2) [32]. The measurements for the PLA-only and PLA continuous basalt fibre composites were obtained based on in-house measurements [15].

The PLA-only parts had a $\tau_{ILSS}$ of 14.6 MPa (±0.3), and they exhibited a failure mode by tensile fracture with little or no shearing between the layers. The ILSS strength of the PLA-SSF 0.22 mm layer height prints was found to be 28.5 MPa (±2.0), achieving a twofold improvement in ILSS strength. Factors which were likely to influence the performance of the composite are the adhesion between the steel fibres and the polymer, the level of impregnation of the PLA within the SSF bundle and the quantity of SSF in the sample along with the level of porosity.

**Figure 9:**
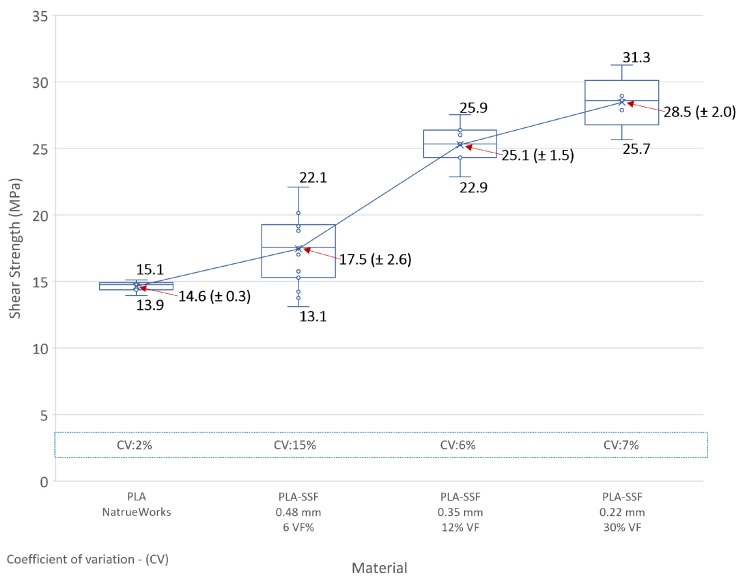
Interlaminar shear strength results for the 0.22, 0.35 and 0.48 mm layer heights investigated with statistical analysis.

With respect to the report from the literature on the ILSS value for PLA-cGF included in Figure 8, no details were provided on the fibre dimensions used to produce the 1 mm diameter filament, which were used for this print [32]. The composite in this case was manufactured from a PLA solution within which the fibre was immersed. In the case of the ILSS test samples, they contained a 4.8 wt% fibre content. A more detailed statistical analysis of the PLA-SSF ILSS results from the current study is plotted in Figure 9, which includes the corresponding coefficient of variation (CV) for the sample sets. As expected for the 0.48 mm layer print height samples, a relatively high level of variation in the ILSS results was obtained (CV of 15%). In the case of the 0.35 mm and 0.22 mm layer height prints, the coefficient of variation was in the range of 5–10%.

**Figure 10:**
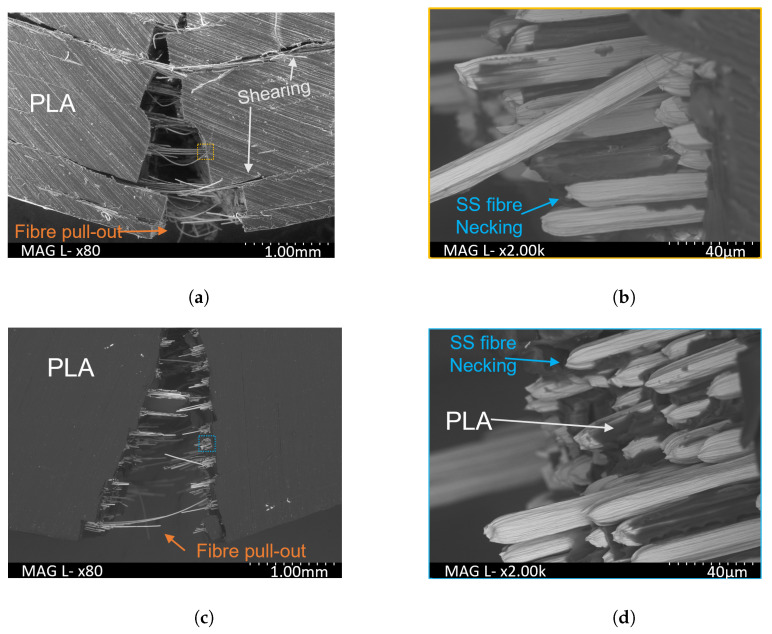
Examination of PLA-SSF composites after ILSS testing. (**a**) Fractured composite printed with a layer height of 0.35 mm, indicating interlaminar shearing and tensile fracture. The yellow dashed square indicates the region at higher magnification in (**b**). (**b**) The fibre pull-out and necking for this sample given in a higher magnification. (**c**,**d**) Corresponding images for a composite printed with a layer height of 0.22 mm, demonstrating similar fracturing along with fibre pull-out and SSF necking. The dashed blue square in (**c**) indicates the higher magnification image in (**d**).

SEM examination of the fractured samples after ILSS testing was used to help evaluate the mode of failure which, as illustrated in Figure 10a, was found to be due to tensile fracturing with interlaminar shearing [61]. The crack propagation moved through each layer, shearing from the next layer’s interface to the next tensile fracture’s initiation point, resulting in a jagged failure crack [63]. Also illustrated are the individual SS fibre pull-out, SS fibre necking and bounce back. The first one is where a fibre pulls out of the matrix at the point of failure. The necking of individual fractured fibres is shown in Figure 10b, where a portion of the SSF fibres which fractured close to the PLA’s fractured surface and others had been pulled out of the matrix before failure.

Comparing the fractured 0.22 and 0.35 mm print composites after ILSS testing demonstrated that the former presented with less fibre pull-out and crack propagation through the layers. Observing the crack for the 0.22 mm sample indicated that there was a lower level of shearing between the layer interfaces, indicating a stiffer composite matrix.

#### 3.3.2. Tensile Properties

**Figure 11:**
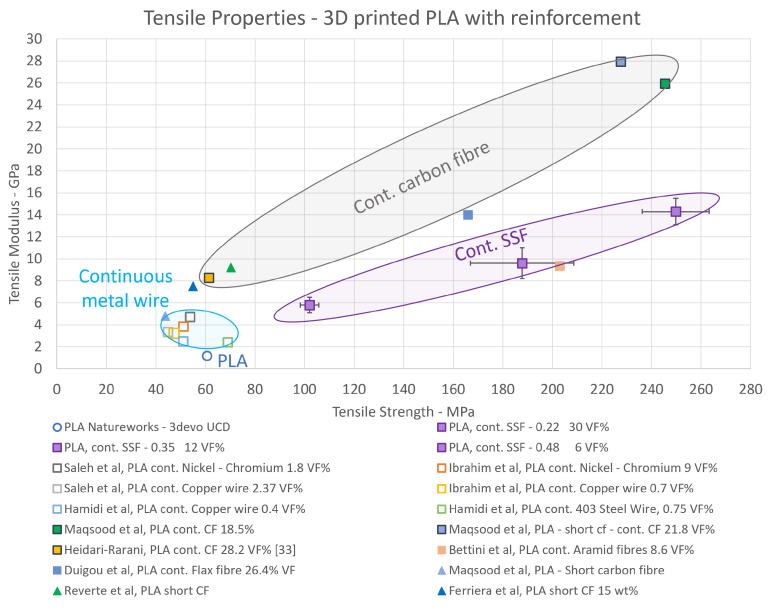
Tensile properties of PLA composite samples printed at the four print head heights in this study (purple squares), with examples of metal- and carbon fibre-reinforced composites reported in the literature [17,19,20,30,33,36,41,44,64].

The tensile performance results for both the PLA and the PLA-SSF are plotted in Figure 11. Also included in this figure are the results reported for a number of other authors who investigated 3D-printed PLA composites, including studies involving natural fibres, metals wires and carbon fibres (continuous and short) [17,18,19,20].

The 3D-printed tensile sample set at a 0.48 mm layer height resulted in a tensile strength of 102.0 MPa (±3.7) and modulus of 5.8 GPa (±0.7). From characterisation and geometry analysis, the structure had a high level of porosity. The filament diameter of 0.50–0.65 mm was close to the printing layer height with a hatch spacing of 0.6 mm, thus likely yielding poor interline bonding.

The PLA-SSF tensile strength printed at a layer height of 0.22 mm was 249.8 MPa (±13.5), and the tensile modulus was 14.3 GPa (±1.2). These values are four and seven times higher, respectively, compared with those obtained for the non-reinforced polymer. The mechanical results displayed a broadly linear pattern where as the fibre volume fraction in the print increased, there was an associated increase in the composites’ ILLS, tensile strength, and modulus. A summary of the volume fractions and tensile strengths of the 3D-printed PLA composites are tabulated in Table 5.

**Figure 12:**
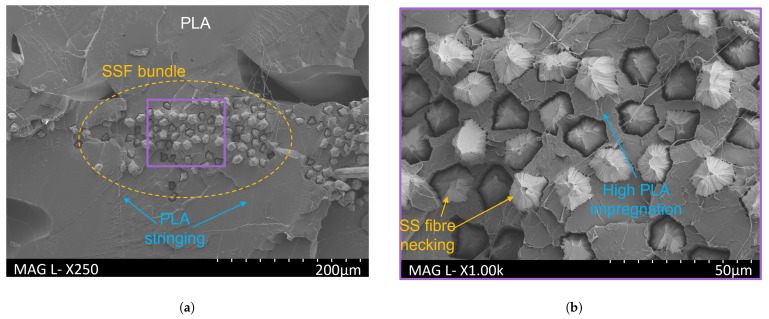
SEM images of PLA-SSF composite (35 mm layer height) after tensile testing. The image on the right is a higher magnification of the region in the purple box in the left image.

SEM images of the cross-section of the 0.35 mm layer height print after tensile testing are given in Figure 12. An SSF bundle is highlighted within the dashed orange oval in Figure 12a. PLA is stiffer than SSF, and due to its brittle failure, it appeared as flat sections on the fractured surface of the composite. As observed for the previous cross-section studies, there was good impregnation of the PLA into the middle of the fibre bundle. As illustrated in Figure 12b, the fibres failed mainly at the fracture face, indicating good inter-facial adhesion between the two materials. All the stainless steel fibres exhibited necking prior to ultimate failure [63]. PLA ‘stringing’, which indicates PLA necking, appeared as a ‘cobweb’-like structure over the fractured surface [65]. The gaps in the PLA observed around a number of the SS fibres were possibly associated with elongation and cross-sectional shrinkage of the steel fibre experienced during necking before ultimate failure. The use of metal fibres increases the contact area between the matrix-reinforcing fibre interface, and this bonding can contribute to improving the mechanical properties [66].

As demonstrated in Figure 11, as the stainless steel fibre volume fraction increased, there was a corresponding enhancement in the tensile properties. This observation is similar to the results obtained by other authors [13]. We note that the PLA-cCF was not found to follow this trend with an increased volume fraction. However, for these composites, the different sizing agents used may also have influenced the composite mechanical properties [33,36]. Table 5 demonstrates that the tensile strength and modulus of the PLA-SSF almost doubled when comparing the samples with layer heights of 0.48 and 0.35 mm, with only a 5% increase in the volume fraction.

These results indicate that as the layer height of the print increased, the impregnation or diffusion of polymer within the SSF bundle was relatively poor due to the shape of the SSF, as discussed in Section 3.1, which thus gave rise to an increase in porosity. The PLA-SSF requires a slow printing speed to achieve polymer solidification, retaining the SSF in position as the print head moves and leading to long print times and potential fibre breakage at higher speeds. A potential advantage of this process and technology is its use in bespoke conductive textiles, along with high impact resistance after-market 3D-printable repairs. A further advantage is sustainable sourcing and potential recyclability for compatible remanufacturing.

The PLA-SSF tensile strength and modulus values obtained were the highest reported for metal-reinforced composites to date. The tensile strengths obtained for the composites with printed layer heights of 0.22 mm were approximately four times higher when compared with continuous spring-back 304 stainless steel wire with a 0.127 mm diameter [44]. When comparing the tensile strengths of the PLA-SSF composite with 7 $V_{f}$% with that reported in the literature for a PLA-nickel chromium wire with 9 $V_{f}$%, the former exhibited strengths which were 50% higher [39]. Amongst the factors which are likely to have contributed to this improvement in mechanical performance in this study are the use of continuous fibres in the prints, the good impregnation of the PLA between the fibres 14 μm in diameter in the 0.15 mm SSF bundle, the relatively low porosity (down to 2%) and the relatively high SSF content ($V_{f}$ up to 30%).

## 4. Conclusions

Filaments of continuous stainless steel fibre bundles within a polylactic acid (PLA) polymer were fabricated using a laboratory-scale extrusion system. By systematically controlling the 3D printing conditions, along with the use of a novel ‘polymer pressure vent’ within the printer nozzle, 3D-printed composites with fibre volume fractions between 4 and 30% were achieved. Good impregnation and adhesion of the PLA matrix into the stainless steel fibre were found based on CT analysis, with the porosity of the resulting composites being in the range of 2–21%. The interlaminar shear strength (τILSS) of the PLA-SSF with a volume fraction of 30% was found to be 28.5 MPa (±2.0), which was twice that of the PLA-only parts. Both the interlaminar shear strength and tensile strength properties of the composites were found to increase significantly as the stainless steel volume fraction ($V_{f}$) increased from 6 to 30%. The PLA-SSF composites exhibited tensile strengths of up to 249.8 MPa (±13.5), along with tensile modulus values of 14.3 GPa (±1.2). The tensile strengths obtained with the highest stainless steel $V_{f}$ in this study were approximately four times higher than those reported for other printed metal fibre-reinforced composites in the literature.

## Figures and Tables

**Table 1:** EDAX analysis elemental composition of the stainless steel fibre.

Element	Fe	Cr	Ni	Mo	Si	Al
Weight %	69.6	17.9	5.6	2.1	0.5	0.4

**Table 2:** The 3devo filament maker processing parameters. Note that $T_{1}$–$T_{4}$ relate to the extrusion barrel heater temperatures, as indicated in Figure 2a.

Filament	Filament	Fans	Extruder	$T_{1}$	$T_{2}$	$T_{3}$	$T_{4}$
	**Diameter**	**(%)**	**Speed**	**(°C)**	**(°C)**	**(°C)**	**(°C)**
	**(mm)**		**(rpm)**				
PLA-SSF	0.90	45	2.7	177	187	186	177
PLA-SSF	0.60–0.70	20	2.6	177	187	186	170
PLA-SSF	0.50–0.65	33	2.2	179	186	193	191

**Table 3:** PLA-SSF 3D print parameters.

Sample Set	Hatch	Filament	Speed	T${}_{\mathrm{nozzle}}$	T${}_{\mathrm{bed}}$
**Layer Height (h)**	**Spacing (w)**	**Diameter**	**(mm/s)**	**(°C)**	**(°C)**
**(mm)**	**(mm)**	**(mm)**			
0.48	0.60	0.50–0.65	3	209	40
0.35	0.40	0.50–0.65	6	209	40
0.22	0.40	0.50–0.65	4	215	55

**Table 4:** Typical volume fraction and porosity results obtained for the printed PLA-SSF composites obtained using μCT analysis.

Sample Set	Volume	Porosity
**Layer Height**	**Fraction ($V_{f}$)**	**(%)**
**(mm)**	**(%)**	
0.48	6	21
0.35	12	6
0.22	30	2

**Table 5:** PLA reinforcement materials, volume fractions and tensile properties.

PLA Reinforcement	$V_{f}$ (%)	Tensile Strength (MPa)	Reference
Continuous Stainless steel fibre bundle 0.22 mm	30	249.8	This work
Continuous Stainless steel fibre bundle 0.35 mm	12	187.8	This work
Continuous Stainless steel fibre bundle 0.48 mm	7	102.0	This work
Continuous Nickel-Chromium wire			
Wire diameter = 75 μm	9.0	51.2	[30]
Continuous nickel-chromium wire			
Wire diameter = 75 μm	1.8	53.8	[19]
Continuous copper wire			
Wire diameter = 75 μm	2.4	44.9	[19]
Continuous copper wire			
Wire diameter = 75 μm	0.7	47.5	[30]
Continuous copper wire			
Wire diameter = 0.127 mm	0.4	59.0	[44]
Continuous spring-back 304 stainless steel wire			
Wire diameter = 0.1778 mm	0.8	61.0	[44]
Continuous carbon fibre	18.5	245.4	[36]
Continuous carbon fibre, (short CF PLA matrix)	21.8	227.6	[36]
Continuous carbon fibre	28.2	61.4	[33]
Continuous carbon fibre	34.0	80.0	[34]
Continuous aramid fibre	8.6	203	[41]
Continuous flax fibre	26.4	166.0	[20]
Short carbon fibre, ColorFabb XT-CF20	20 wt%	43.6	[36]
Short carbon fibre, CarbonX™ filament	-	70.3	[17]
Short carbon fibre, Proto-Pasta filament	15 wt%	53.4	[64]

## Data Availability

The data presented in this study are available upon request from the corresponding author.

## Abbreviations

The following abbreviations are used in this manuscript:

3D	Three-dimensional
°C	Degrees Centigrade
*b*	Specimen width
*h*	Specimen thickness
kN	Kilonewton
μCT	Micro computed tomography
μm	Micrometer
mm	Millimeter
mm/s	Millimeters Per second
wt%	Weight percent
$F_{tu}$	Tensile strength
$P_{m}$ or $P_{max}$	Maximum load or failure
rpm	Revolutions per minute
$T_{1}$	1st Extrusion barrel heater temperature
$T_{2}$	2st Extrusion barrel heater temperature
$T_{3}$	3st Extrusion barrel heater temperature
$T_{4}$	4st Extrusion barrel heater temperature
ABS	Acrylonitrile butadiene styrene
CAD	Computer-aided design
cCF	Continuous carbon fibre
EDAX	Energy-dispersive X-ray spectroscopy
ILSS or $\tau_{ILSS}$	Interlaminar shear strength
L	Loop 3D print geometry
GPa	Gigapascal
MEX	Material extrusion
MPa	Megapascal
PC	Polyamide or nylon
PC	Polycarbonate
PLA	Polylactic acid
PLA-cCF	Polylactic acid containing continuous carbon fibre
PLA-cGF	Polylactic acid containing continuous glass fibre
PLA-SCF-cCF	Polylactic acid containing short carbon fibres and continuous carbon fibre
PLA-SSF	Polylactic acid containing continuous stainless steel fibre bundle composite
SEM	Scanning electron 255 microscope
SSF	316 L stainless steel fibre bundle
ATL	Stereolithography
TEX	Decitex
$V_{f}$	Volume fraction
$V_{SSF}$	Volume of continuous stainless steel fibre
$V_{T}$	Overall volume
